# Increased susceptibility of human endothelial cells to infections by SARS-CoV-2 variants

**DOI:** 10.1007/s00395-021-00882-8

**Published:** 2021-07-05

**Authors:** Julian U. G. Wagner, Denisa Bojkova, Mariana Shumliakivska, Guillermo Luxán, Luka Nicin, Galip S. Aslan, Hendrik Milting, Joshua D. Kandler, Andreas Dendorfer, Andreas W. Heumueller, Ingrid Fleming, Sofia-Iris Bibli, Tobias Jakobi, Christoph Dieterich, Andreas M. Zeiher, Sandra Ciesek, Jindrich Cinatl, Stefanie Dimmeler

**Affiliations:** 1 Institute for Cardiovascular Regeneration, Centre of Molecular Medicine, Goethe University Frankfurt, Theodor Stern Kai 7, 60590 Frankfurt, Germany; 2 Institute of Medical Virology, University Frankfurt, Frankfurt, Germany; 3 German Center for Cardiovascular Research (DZHK), Partner Site Rhein-Main, Frankfurt, Germany; 4 Cardiopulmonary Institute (CPI), Frankfurt, Germany; 5 Clinic for Thoracic and Cardiovascular Surgery, Bad Oeyenhausen, Germany; 6 Walter-Brendel-Centre, Hospital of the Ludwig-Maximilians-University München, Munich, Germany; 7 Institute for Vascular Signalling, Centre for Molecular Medicine, Goethe University, Frankfurt am Main, Germany; 8 Department of Internal Medicine and the Center for Translational Cardiovascular Research, University of Arizona, 475 N. 5th Street, Phoenix, AZ 85004 USA; 9 Klaus Tschira Institute for Integrative Computational Cardiology, University Hospital Heidelberg, Heidelberg, Germany; 10 Department of Cardiology, University Frankfurt, Frankfurt, Germany; 11 Fraunhofer Institute for Molecular Biology and Applied Ecology (IME), Branch Translational Medicine und Pharmacology, Frankfurt, Germany; 12 German Centre for Infection Research (DZIF), External Partner Site Frankfurt, Frankfurt, Germany

**Keywords:** Endothelial cells, SARS-CoV-2, Virus trapping, ER stress

## Abstract

Coronavirus disease 2019 (COVID-19) spawned a global health crisis in late 2019 and is caused by the novel coronavirus SARS-CoV-2. SARS-CoV-2 infection can lead to elevated markers of endothelial dysfunction associated with higher risk of mortality. It is unclear whether endothelial dysfunction is caused by direct infection of endothelial cells or is mainly secondary to inflammation. Here, we investigate whether different types of endothelial cells are susceptible to SARS-CoV-2. Human endothelial cells from different vascular beds including umbilical vein endothelial cells, coronary artery endothelial cells (HCAEC), cardiac and lung microvascular endothelial cells, or pulmonary arterial cells were inoculated in vitro with SARS-CoV-2. Viral spike protein was only detected in HCAECs after SARS-CoV-2 infection but not in the other endothelial cells tested. Consistently, only HCAEC expressed the SARS-CoV-2 receptor angiotensin-converting enzyme 2 (ACE2), required for virus infection. Infection with the SARS-CoV-2 variants B.1.1.7, B.1.351, and P.2 resulted in significantly higher levels of viral spike protein. Despite this, no intracellular double-stranded viral RNA was detected and the supernatant did not contain infectious virus. Analysis of the cellular distribution of the spike protein revealed that it co-localized with endosomal calnexin. SARS-CoV-2 infection did induce the ER stress gene EDEM1, which is responsible for clearance of misfolded proteins from the ER. Whereas the wild type of SARS-CoV-2 did not induce cytotoxic or pro-inflammatory effects, the variant B.1.1.7 reduced the HCAEC cell number. Of the different tested endothelial cells, HCAECs showed highest viral uptake but did not promote virus replication. Effects on cell number were only observed after infection with the variant B.1.1.7, suggesting that endothelial protection may be particularly important in patients infected with this variant.

**Supplementary Information:**

The online version contains supplementary material available at 10.1007/s00395-021-00882-8.

## Introduction

Severe acute respiratory syndrome coronavirus 2 (SARS-CoV-2) causes the coronavirus disease 2019 (COVID-19) that in the last year gave rise to a global pandemic. SARS-CoV-2 primarily invades alveolar epithelial cells and causes acute respiratory distress syndrome. However, increasing evidence indicates that endothelial cell dysfunction and vascular events are major complications of the disease. Indeed, vascular inflammation, barrier defects leading to tissue edema, activation of disseminated intravascular coagulation and microthrombi were reported in moderate to severe COVID-19 cases [3, 8, 14, 16, 27, 28, 30]. In addition, pre-existing impaired endothelial function, i.e., in diabetes mellitus patients and underlying vascular pathologies were shown to worsen clinical outcome of COVID-19 [3, 8, 14, 16, 27, 28, 30]. Whether vascular complications can be attributed to a systemic inflammatory response or are a direct consequence of the viral infection of endothelial cells is currently under debate. To-date, SARS-CoV-2 has been reported to directly infect vascular organoids in vitro [19] and first case studies reported endothelial infection in glomerular capillary loops, skin lesions [8, 10, 30], as well as provided evidences for endotheliitis in COVID-19 patients [17, 30]. However, the expression of the putative SARS-CoV-2 receptor, i.e., angiotensin-converting enzyme 2 (ACE2) is low in endothelial cells compared to mural cells and recent studies suggest that endothelial cells may not be the primary target of SARS-CoV-2 in the vascular wall [11]. This would imply that the endothelium might be affected independently of direct viral action during the course of the disease, whereby the cytokine storm syndrome associated with elevated levels of pro-inflammatory cytokines such as IL-1β, IL-6, and TNFα may cause the loss of anti-thrombotic and anti-inflammatory functions of endothelial cells [23, 28].

To gain insight into whether endothelial cells are a primary target of SARS-CoV-2, we studied human endothelial cells derived from several different vascular beds in vitro.

## Materials and methods

### Cells and cardiac tissues

Human umbilical vein endothelial cells (HUVEC; CC-2935), human coronary artery endothelial cells (HCAEC; CC-2585), human cardiac microvascular endothelial cells (HCMVEC; CC-7030), and human lung microvascular endothelial cells (HLMVEC; CC-2527), and human pulmonary arterial cells isolated from diabetics (D-HPAEC; CC-2924) were purchased from Lonza and cultured in endothelial basal medium (EBM; CC-3156, Lonza) supplemented with 10% fetal calf serum (FCS; 4133, Invitrogen), amphotericin-B, ascorbic acid, bovine brain extract, endothelial growth factor, gentamycin sulfate, and hydrocortisone (EGM-Bullet Kit; CC-3124, Lonza) or EBM-2 supplemented with 10% FCS, hydrocortisone, FGF, VEGF, R3-IGF, ascorbic acid, EGF and GA-1000 (EGM-2-Bullet kit; CC-3162, Lonza) at humidified atmosphere, at 37 °C/5% CO_2_.

Primary human umbilical vein endothelial cells were isolated and purified using CD144 antibody-coated magnetic beads (Dynal Biotech, Hamburg, Germany) and cultured as reported previously [6]. The human umbilical cords were obtained from local hospitals in Frankfurt am Main and the use of human material in this study conforms to the principles outlined in the Declaration of Helsinki. The isolation of human cells was approved by the ethics committee at the Goethe University in Frankfurt am Main. HUVEC were cultured in endothelial basal medium (EBM; CC-3156, Lonza) as mentioned above for the commercial HUVEC.

Living human heart slices were generated and cultured as recently described [9]. Samples of left ventricular myocardium were obtained from failing hearts during transplantation in the Clinic of Thoracic and Cardiovascular Surgery, Heart and Diabetes Center, Bad Oeynhausen, Germany. The procedure has been approved by the institutional ethics board, and patients have provided informed consent to the scientific use of the explanted tissue. In brief, heart slices were generated from the explanted failing human myocardium by cutting 300-µm-thick vibratome sections. Slices were mounted and cultured in biomimetic culture chambers in a standard incubator (37 °C, 5% CO_2_, 20% O_2,_ 80% humidity) [9]. Pacing was performed at 0.5 Hz with bipolar 50 mA pulses comprised of 1 ms charging and discharging pulses separated by a 1 ms interval. Slices were cultured in Medium 199 supplemented with penicillin/streptomycin, insulin/transferrin/selenite and 50 µM 2-Mercaptoethanol. Medium was exchanged in part (1.6 ml of 2.4 ml total volume in each biomimetic cultivation chamber) at 36–48 h intervals.

### Infection

SARS-CoV-2 (strains: D614, G614, B.1.1.7, B.1.351, and P.2) were isolated and propagated in CaCo2 cells as previously described [4, 12]. For infection of endothelial cells, the viral stock was diluted to the desired MOI (multiplicity of infection) in the respective medium supplemented with 1% FCS and incubated for 2 h. Then, the medium was changed to the respective culture media (see above). Five days after infection, endothelial cells were fixed in 4% paraformaldehyde (PFA) for 10 min or lysed for RNA isolation. CaCo2 cells were fixed after 24 h. Living human heart slices were incubated with 200 µl of viral stock (1.10^7^ TCID 50/ml) for 3–5 days using the above-mentioned medium without 2-Mercaptoethanol.

For experiments that mimic pro-inflammatory conditions, endothelial cells were treated for 24 h with 30 ng/ml TNFα (210-TA-005, R&D Systems), 10 ng/ml IFNβ (8499-IF-010, R&D Systems) or 10 ng/ml IFNγ (285-IF-100, R&D Systems) prior to viral infection.

To block proteasomal degradation, cells were incubated with the virus together with 10 µM MG132 (M7449, Sigma-Aldrich; solved in DMSO) for 24 h.

To inhibit viral infection, HCAECs pre-incubated with 10 µM chloroquine (PHR1258, Sigma-Aldrich), 1 µM (in DMSO) cathepsin inhibitor *N*-Acetyl-l-leucyl-l-leucyl-l-methional (ALLM; 0384, Tocris) or 20 µM Furin Inhibitor I (344390-AMG, Merck). 5 µg/ml human recombinant ACE2 (933-ZN, R&D Systems) was mixed with the virus and incubated for 30 min, prior to infection. Cells were fixed 3 days after infection.

### Immunofluorescence labeling

Cells were fixed with 4% PFA and were permeabilized with 0.1% Triton X-100/PBS for 10 min. Cells were blocked with blocking solution (5% donkey serum in PBS) for 1 h at RT. Primary antibodies were incubated in blocking solution overnight at 4 °C. After four 5-min washes with PBS, secondary antibodies and DAPI were incubated for 1 h in blocking solution. Finally, two 5-min washes of PBS were performed before cell observation.

Heart slices were fixed in 4% HistoFix (P087.4, Carl Roth GmbH) for 24 h at 4 °C. Slices were transferred to a series of ascending sucrose concentration: 4% sucrose in PBS (1 h, 4 °C), 15% sucrose in PBS (4 h, 4 °C) and finally 30% sucrose in PBS (overnight, 4 °C). The day after, slices were washed twice for 30 min with 100 mM glycine at 4 °C and once with PBS for 30 min at 4 °C. Cardiac slices were permeabilized with 1% Triton X-100 in PBS overnight at 4 °C. Slices were then washed three times with PBS for 30 min at 4 °C and blocked with blocking solution (3% BSA in 0.3% Triton X-100) overnight at 4 °C. Slices were washed three times in 0.3% Triton X-100 and incubated with primary antibodies that were diluted in blocking solution (overnight, 4 °C). Slices were again washed trice with 0.3% Triton X-100/PBS (30 min each) and incubated with the secondary antibodies that were diluted in PBS (overnight, 4 °C). Finally, slices were again washed trice with 0.3% Triton X-100/PBS before mounting (see Suppl. Tab. 1 for detailed antibody information).

### RT-qPCR and gel electrophoresis

Total RNA was isolated using RNeasy Mini Kit (217004, Qiagen) according to the manufacturer’s instructions including an on-column DNase I digestion step (79254, Qiagen). Reverse transcription was performed using 500 ng RNA, random hexamers and MuLV reverse transcriptase (N8080018, Thermo Fisher). Fast SYBR Green qPCR were carried out by StepOnePlus real-time PCR systems (4385617, Thermo Fisher). RPLP0 amplification was used for data normalization. Relative expression levels were calculated by 2^‒ΔCt^. Primer sequences are provided in Suppl. Table 2. PCR products were visualized on a 1.5% agarose gel in 1xTAE buffer and visualized with Midori Green Advance (617004, Biozym Scientific GmbH).

### Quantification of virus titer in cell culture supernatants

Supernatants from infected endothelial cells were collected 5 days post-infection. Confluent layers of CaCo_2_ cells in 96-well plates were infected with serially diluted supernatants. Cytopathogenic effect (CPE) was assessed visually 48 h after infection. The infectious titer was determined as TCID50/ml.

### Single-nuclei RNA-sequencing analysis

Single-nuclei RNA-sequencing data from human cardiac samples were derived from a previously published data set [21]. Data were analyzed with the Seurat (v3) package.

### Statistical analysis

Data are represented as mean and error bars indicate standard error of the mean (SEM). Data were statistically assessed for Gaussian distribution using Kolmogorov–Smirnov and Shapiro–Wilk test. For comparison of two groups, statistical power was determined using two-tailed, unpaired *t* test. For multiple comparisons, ordinary one-way ANOVA with a post hoc Dunnett’s or Turkey’s multiple comparison was used.

## Results

### Infection of different endothelial cells by SARS-CoV-2

**Fig. 1:**
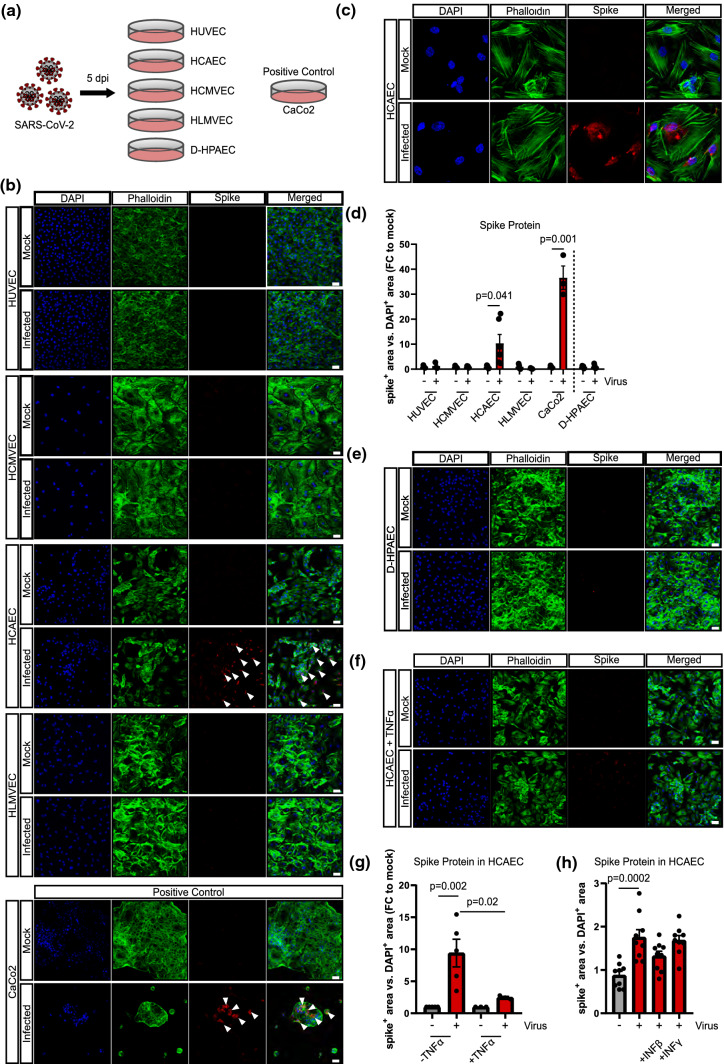
Human coronary artery endothelial cells are permissive for SARS-CoV-2. **a** Schematic experimental setup. **b** Human umbilical vein endothelial cells (HUVEC), human coronary artery endothelial cells (HCAEC), human cardiac microvascular endothelial cells (HCMVEC), and human lung microvascular endothelial cells (HLMVEC) were commercially purchased and were infected with SARS-CoV-2 (MOI = 1) for 2 h. Cells were cultured for 5 days. Human colon carcinoma cells (CaCo2) were used as positive control and harvested after 1 day. Spike protein (red) was detected using the rabbit-SARS-CoV-2 Spike primary antibody (indicated by white arrows) and cells were counterstained for DAPI (blue) and phalloidin (green). **c** Representative high magnification image of HCAECs experiments shown in **b**. **d** Quantification of data shown in B (n = 6 for HCAEC, n = 3 all other ECs). **e** Human lung pulmonary arterial cells isolated from diabetics (D-HPAEC) were commercially purchased and were infected with SARS-CoV-2 (MOI = 1) for 2 h. Cells were cultured for 5 days. Spike protein (red) was detected using the rabbit-SARS-CoV-2 Spike primary antibody and cells were counterstained for DAPI (blue) and phalloidin (green). *n* = 3. **f** HCAEC were treated with 30 ng/mL TNFα prior to SARS-CoV-2 inoculation (as described for panel **b**) *n* = 3. Cells were cultured for 5 days. Spike protein (red) was detected using the rabbit-SARS-CoV-2 Spike primary antibody and cells were counterstained for DAPI (blue) and phalloidin (green). **g** Quantification of data shown in **b** and **f**. **h** HCAEC were infected with SARS-CoV-2 isolates in the presence and absence of 10 ng/ml human recombinant interferon beta (IFNβ) or interferon gamma (IFNγ). Cells were cultured for 5 days and fixed with 4% PFA. Spike protein (red) was detected using the rabbit-SARS-CoV-2 Spike primary antibody (provided by Hölzel) and cells were counterstained for DAPI (blue) and phalloidin (green). Data are shown as mean and error bars indicate the standard error of the mean (SEM). After passing normality tests, data were statistically accessed using an unpaired, two-tailed *T* test to compare mock treated cells to their respective infected counterpart (**d**) or using a one-way ANOVA test with a post hoc Turkey’s test (**g**, **h**). Scale bars = 50 µm

Human umbilical vein endothelial cells (HUVEC), human coronary artery endothelial cells (HCAEC), human cardiac microvascular endothelial cells (HCMVEC) and human lung microvascular endothelial cells (HLMVEC) were incubated with isolated SARS-CoV-2 [4] for 2 h and viral infection was measured 5 days post-infection by detection of intracellular viral spike protein (Fig. 1a). Spike protein was only weakly detected in HCAEC and not in any of the other endothelial cells (Fig. 1b–d), even when extending the duration of infection to 24 h (data not shown). The activity of the viral strain used [5] was evidenced by its ability to infect CaCo2 cells (Fig. 1b, d). Since we used expanded endothelial cell cultures that might change their gene expression during passaging, the ability of the virus to infect freshly isolated HUVEC was also tested. In line with the results obtained with commercially available HUVEC, no viral spike protein could be detected following incubation with SARS-CoV-2 (Suppl. Fig. 1a–c). Of note, endogenous degradation pathways might also prevent significant virus accumulation in HCAEC. To test this assumption, we treated HCAEC with the proteasomal inhibitor MG132. MG132 significantly increased spike protein accumulation suggesting that indeed endogenous degradation pathways limit viral protein accumulation (Suppl. Fig. 2).

Since diabetes mellitus is a risk factor for complicated courses of COVID-19, we additionally infected pulmonary artery endothelial cells from subjects with diabetes (D-HPAEC). However, no spike protein was detected (Fig. 1d, e). In addition, we investigated whether or not a pro-inflammatory environment could sensitize endothelial cells to SARS-CoV-2 infection. To mimic a pro-inflammatory environment as it would occur in COVID-19 patients, we pre-incubated endothelial cells with TNF-α or interferons for 24 h prior to infection. However, the pre-exposure of endothelial cells to TNF-α, IFNβ or IFNγ did not change the levels of spike protein in any of the endothelial cells studied (Fig. 1f–h and Suppl. Fig. 2). Rather, the inflammatory cytokine TNF-α reduced spike protein levels in SARS-CoV-2 infected HCAECs (Fig. 1f, g, Suppl. Fig. 3). Given the discussion, that hydrocortisone may be antiviral [17] and given the fact that the used media contain hydrocortisone, we repeated the SARS-CoV-2 infection experiments in hydrocortisone-depleted media. However, no differences in spike detection were found in the presence and absence of hydrocortisone (data not shown).

**Fig. 2:**
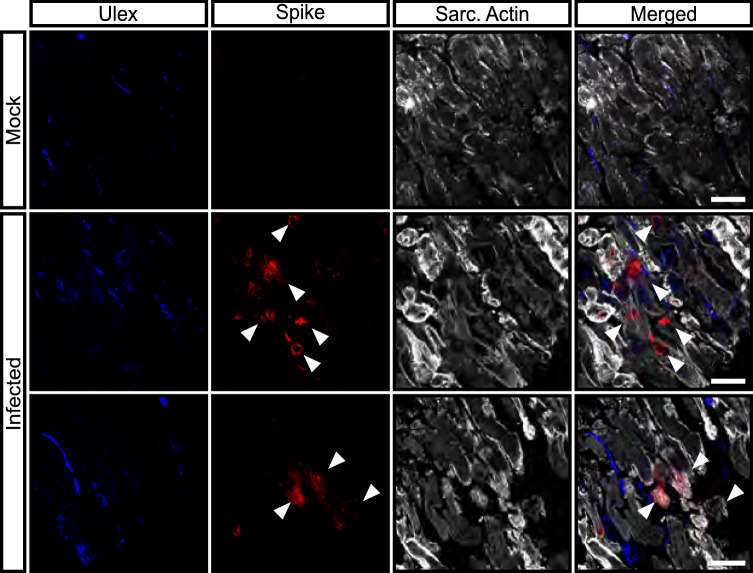
SARS-CoV-2 infection in living human heart slices. Spike protein (red) was detected using the rabbit-SARS-CoV-2 Spike primary antibody (indicated by white arrows) and slices were counterstained for DAPI (blue) and sarcomeric-actin (grey). Shown are one representative image for mock infected slices and two representative images for infected heart slices. Scale bars = 50 µm

We additionally explored whether the cardiac microvascular endothelium can be infected in human heart slides ex vivo. However, only cardiomyocytes and other interstitial cells were positive for spike protein (Fig. 2, white arrows). There was no endothelial cell found to be positive for spike protein. Together these data suggest that only coronary artery endothelial cells but, not cardiac microvascular or pulmonary endothelial cells are susceptible to SARS-CoV-2 infection in vitro*.*

### Expression of putative SARS-CoV-2 receptors in human endothelial cells

**Fig. 3:**
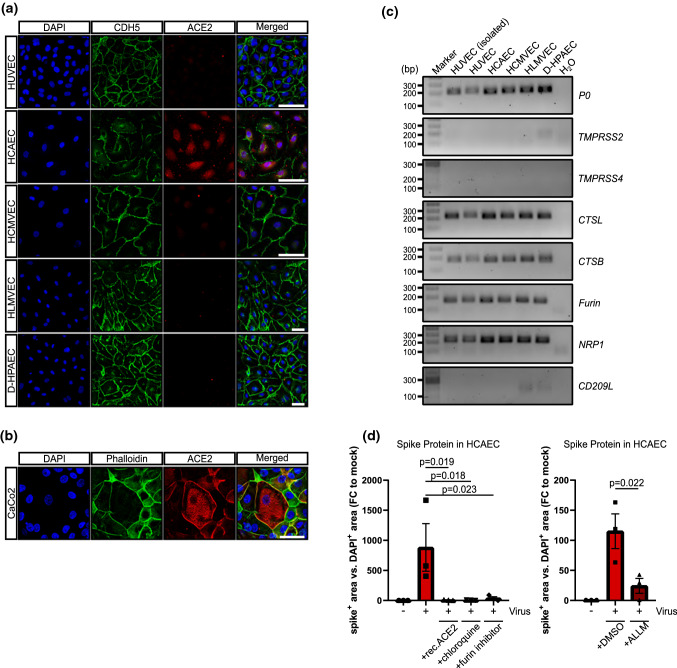
SARS-CoV-2 receptor expression in different endothelial cell cultures. **a**, **b** HUVEC, HCMVEC, HCAEC, HLMVEC, D-HPAEC and CaCo2 cells were seeded at 80% confluence and were stained 1 day after against ACE2 (red). DAPI (blue) and CDH5/phalloidin (green) served as counter staining. Scale bars = 50 µm. **c** RT-PCR products of *TMPRSS2, TMPRSS4*, *CTSL*, *CTSB*, *FURIN, NRP1* and *CD209L* in cultured endothelial cells and freshly isolated HUVEC. PCR products were run on a 1.5% agarose gel in 1 × TAE buffer. **d** Spike protein quantification in HCAEC, 3 days after infection. Cells were incubated with 10 µM chloroquine, 1 µM (in DMSO) cathepsin inhibitor *N*-Acetyl-l-leucyl-l-leucyl-l-methional (ALLM) and 20 µM FURIN-inhibitor I. 5 µg/ml of human recombinant ACE2 was mixed with the virus and incubated for 30 min, prior to infection. Cells were fixed and stained against viral spike protein. Experiments were conducted in triplicate. Data are shown as mean and error bars indicate SEM. Data were statistically assessed using a one-way ANOVA test with a post hoc Dunnett’s test

Next, we determined whether the different susceptibility of endothelial cell types to SARS-CoV-2 infection might be explained by expression of putative SARS-CoV-2 receptors. The well-established co-receptor ACE2 was expressed by HCAEC but not by any of the other endothelial cells (Fig. 3a, Suppl. Fig. 1a). However, unlike CaCo2 cells, where ACE2 proteins was concentrated at the cell membrane, in HCAECs ACE2 was largely present in the perinuclear area with little or no protein detectable at the plasma membrane (Fig. 3a, b). Expression of the mRNA encoding the protease TMPRSS2, which was shown to act as an activator of SARS-CoV-2 entry, was below detection level in all of the endothelial cells studied (CT-values of water control: 36, TMPRSS2: 37.8 ± 0.7 in endothelial cells compared to 22 ± 0.2 in CaCo2 cells). Other proteases known to be involved in SARS-CoV-2 uptake, such as cathepsin B and cathepsin L, were expressed in the endothelial cells tested (Fig. 3c). Neuropilin-1 (NRP1) was recently reported to bind to Furin-cleaved substrates and to potentiate SARS-CoV-2 infectivity [7]. NRP1 and FURIN were clearly expressed in heart tissue as determined by single nucleus RNA sequencing (Suppl. Fig. 4) and in all of the cultured endothelial cells (Fig. 3c). CD209L was reported to be a further receptor for SARS-CoV-2 in epithelial and endothelial cells [1], and surprisingly, was weakly expressed by HLMVEC and D-HPAEC only (Fig. 3c). Interestingly, bulk RNA sequencing analysis of human aortic endothelial cells, HCMVEC and HUVEC showed elevated levels of ACE2, CTSL and NRP in aortic endothelial cells, which may explain why arterial or coronary endothelial cells might be more prone to SARS-CoV-2 infection (Suppl. Fig. 5). To assess the involvement of these proteins in SARS-CoV-2 entry, SARS-CoV-2 infected HCAECs were co-incubated with human recombinant ACE2, FURIN-inhibitor I, chloroquine and the non-selective cathepsin inhibitor ALLM. Interestingly, 3 days post-infection, we found a significant reduction in spike protein staining under all of these conditions (Fig. 3d).

### Effects of SARS-CoV-2 infection on HCAECs

**Fig. 4:**
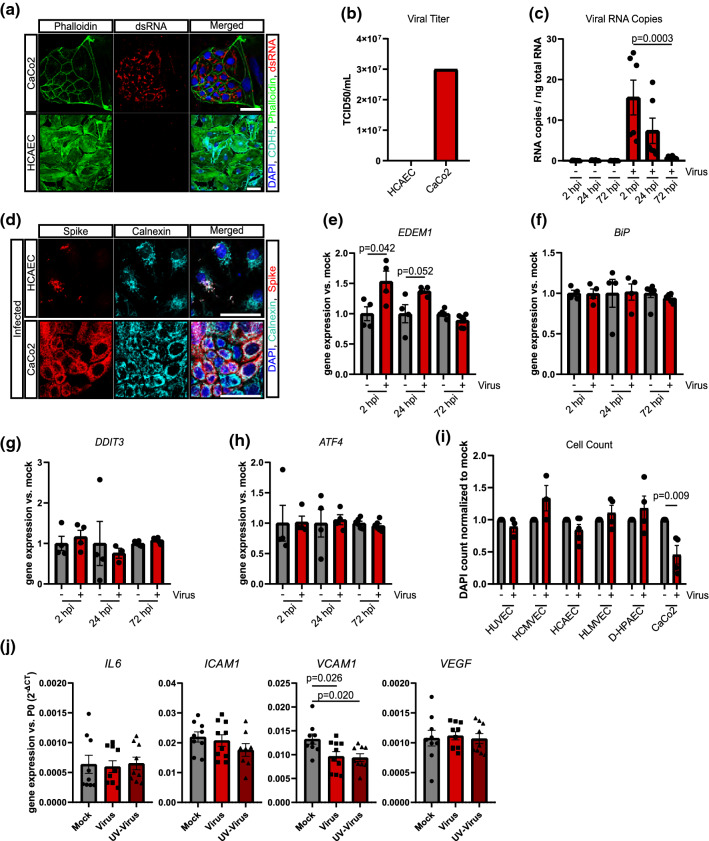
SARS-CoV-2 does not replicate in HCAEC. **a** Detection of double-strand viral RNA (red) in infected CaCo2 cells as positive control (upper panel) and infected HCAEC (lower panel). CDH5 (cyano), Phalloidin (green) and DAPI (blue) served as counterstain. **b** Infectious virus in supernatants from infected HCAEC and CaCo2 cells was determined by titration in CaCo2 cells 48 h post-infection *n* = 3. **c** Detection of viral RNA copies per ng total RNA lysate in HCAEC 2 h, 24 h and 72 h post-infection (hpi) *n* = 3. **d** Immunostaining of calnexin (cyano) and spike protein (red) in HCAEC (5 dpi) and CaCo2 cells (1 dpi). **e–h** mRNA expression of *EDEM1*, *BiP*, *DDIT3* and *ATF4* in HCAEC 2 h, 24 h and 72 h post-infection *n* = 4 (2 hpi, 24 hpi) and *n* = 6 (72 hpi). **i** DAPI-positive cells were counted in experiments shown in panel **a** *n* = 3 and *n* = 6 (HCAEC). **j** mRNA expression of *IL-6*, *ICAM1*, *VCAM1* and *VEGF* was determined by RT-qPCR *n* = 9. Data are shown as mean and error bars indicate the standard error of the mean (SEM). After passing normality tests, data were statistically accessed using an unpaired, two-tailed *T* test to compare mock treated cells to their respective infected counterpart (**e**–**i**). To compare more than two groups, data were assessed statistically using one-way ANOVA with a post hoc Dunnett’s multiple comparison test (**c**, **j**). Scale bars = 50 µm

Although we detected spike protein in HCAECs after SARS-CoV-2 infection, this does not necessarily document permissive infection of the endothelial cells. Therefore, we further assessed the levels of double-stranded RNA, as a sign of viral RNA synthesis [25, 29], and the presence of infectious virus in the cell supernatant, which would be indicative of viral replication. However, neither double-strand RNA (Fig. 4a) nor infectious virus in HCAEC supernatant could be detected 5 days after SARS-CoV-2 infection (Fig. 4b, Suppl. Table 3). Intracellular RNA copies were only transiently increased during the initial 3 days (Fig. 4c), suggesting that no new virus is generated by the tested endothelial cells and that the spike protein may originated from the virus that was originally taken up.

Indeed, SARS-CoV-2 has been reported to enter cells via receptor-mediated endocytosis [29], and the spike protein detected in HCAECs was localized to endosomal calnexin-positive areas (Fig. 4d). To determine if endosomal localized virus may elicit an ER stress response, we measured various genes known to be induced by ER stress. Interestingly, only EDEM1, which is involved in the clearance of misfolded proteins [18], was transiently up-regulated at day 1 and day 2 post-infection (Fig. 4e), whereas *BiP*, *DDIT3* and *ATF4* mRNA expression were not affected (Fig. 4f–h).

Finally, we assessed putative cytotoxic effects in the SARS-CoV-2-infected endothelial cells. However, we did not find evidence for cytopathic effects in the endothelial cells studied (Fig. 4i, Suppl. Figs. 1d, 3c). Moreover, expression of the inflammatory cytokine IL-6, the adhesion molecule ICAM1 and the pro-angiogenic and permeability inducing VEGF were not changed by SARS-CoV-2 infection. VCAM1 was down-regulated by active and UV-irradiated SARS-CoV-2 virus (Fig. 4j).

### Effects of SARS-CoV-2 variants on HCAECs

**Fig. 5:**
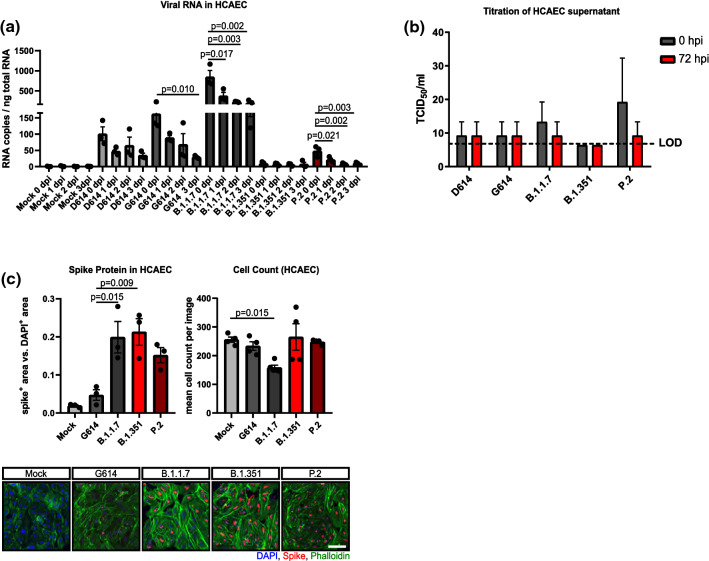
SARS-CoV-2 variants do not replicate in endothelial cells. **a** Detection of viral RNA copies per ng total RNA lysate in HCAEC 0 days, 1 day, 2 days and 3 days post-infection (dpi). Two early isolates (D614, G614) as well as the variants B.1.1.7, B.1.351 and P.2 SARS-CoV-2 variants were used (*n* = 3). **b** Infectious virus in supernatants from infected HCAEC was determined by titration in CaCo2 cells 0 h and 48 h post-infection (hpi) *n* = 3. **c** Immunostaining of spike protein (red) in HCAEC (5 dpi). DAPI (blue) and Phalloidin (green) were used as counter staining. HCAEC were infected with one virus isolate D614 as well as the B.1.1.7, B.1.1.7 and P.2 SARS-CoV-2 variant (*n* = 3). Data are shown as mean and error bars indicate the standard error of the mean (SEM) or standard deviation (SD, panel **b**). After passing normality tests, data were statistically accessed using a one-way ANOVA with a post hoc Turkey’s comparison test (**b**) or post hoc Dunnett’s comparison (**a**, **c**). Scale bars = 50 µm

Recently, we have isolated and characterized several novel SARS-CoV-2 variants including B.1.1.7 (mutations include N501Y and del69/70), B.1.351 (mutations include E484K and N501Y) and P.2 (mutations include E484K in the absence of a N501Y mutation) [31]. Emergence of these variants has raised concerns regarding their host infection capability. We have performed comparative experiments to test whether the novel variants may infect and replicate in HCAECs. Cells were infected with different SARS-CoV-2 variants (D614, G614, B.1.1.7, B.1.351, P.2) at MOI 1. Viral replication was assessed by measurement of viral RNA at different time points post-infection. The production of infectious viral particles was determined by titration of infected cell supernatants on confluent CaCo2 cell layers. Although intracellular viral RNA was detected after infection of HCAECs with the variants, particularly the variant B1.1.7, no increase was observed over time (Fig. 5a), indicating that the virus enters the cell, but does not replicate in the endothelial cells. Consistently, none of the variants produced infectious viral particles (Fig. 5b). These data demonstrate that SARS-CoV-2 variants are not able to replicate in HCAEC. However, we observed enhanced virus uptake in endothelial cells by novel SARS-CoV-2 variants (B.1.1.7, B.1.351, and P.2) in association with increased spike staining compared to early variants (D614 and G614) (Fig. 5c). Interestingly, cell toxicity was not observed, despite B.1.1.7, which significantly reduced HCAEC counts (Fig. 5c).

## Discussion

The results of the current investigation suggest that SARS-CoV-2 does not permissively infect microvascular or venous endothelial cells from different sources. However, HCAEC appear to take up the virus and show positive spike protein in the endosomal compartment. Since no permissive infection was detected in endothelial cells, SARS-CoV-2 might indirectly induce endothelial cell dysfunction via the systemic inflammatory response that causes the observed striking vascular effects in patients with COVID-19.

Interestingly, of all the endothelial cells studied only HCAECs were positive for spike protein after SARS-CoV-2 infection, suggesting different responses of endothelial cells derived from different vascular beds. Endothelial cell heterogeneity and specificity is crucial for the homeostasis of the different organs [2]. In our study, HCAECs show a higher expression of the SARS-CoV-2 receptor ACE2, which may be responsible for mediating the uptake of the virus. HCMVEC, HLMVEC, HUVEC and HPAEC did not express ACE2 and were not permissive for SARS-CoV-2. However, transducing endothelial cells with recombinant ACE2 may enable SARS-CoV-2 infection as shown recently [20], suggesting that the lack of sufficient ACE2 expression might be a limiting factor. Subsequent steps in the viral life cycle appear to be blocked in HCAECs. Since we did not detect double-strand viral RNA in HCAECs upon infection, likely early steps in uncoating of the incoming virus, or endosome-virus membrane fusion, or viral RNA synthesis are haltered in HCAECs. It would be of interest to understand the mechanisms that allow this endothelial cell type to block subsequent virus replication [29]. Interestingly, we observed an early induction of EDEM1, which is known to be involved in clearance of misfolded proteins in the ER [18]. One may speculate that the induction of this gene may be involved in the removal of spike protein limiting further viral activities.

The observation that endothelial cells take up SARS-CoV-2 without propagating the virus was also made previously [22, 32]. However, these studies were performed with stem cell-derived endothelial cells, which may not fully resemble primary cultured lung or cardiac endothelial cells, as used in our study. In addition, one report found no evidence of direct viral infection of vascular endothelial cells in an ex vivo lung culture of one COVID-19 patient [13], which was supported by others [24] and by our own ex vivo heart slice model, where we could not detect spike positive endothelial cells. To our knowledge, this is currently the first study to compare the effect of SARS-CoV-2 variants on endothelial cells. We demonstrate a higher uptake of the three tested virus variants in HCAECs, however, B.1.1.7 was the only variant that affected cell number.

One limitation of the present study and previous reports is that we cannot exclude that endothelial cells in humans might react differently and that the in vitro culture and expansion of endothelial cells change their gene expression and responses. We also tested whether a pro-inflammatory environment would have facilitated virus infection. However, stimulation of endothelial cells with pro-inflammatory cytokines or using endothelial cells cultured from diabetic patients did not augment the SARS-CoV-2 infection in the present in vitro study. Recent findings suggested that inflammatory cytokines contribute to a complicated course of COVID-19 [15]. Therefore, we speculate that pre-treatment of endothelial cells with pro-inflammatory cytokines might sensitize endothelial cells for viral infection. However, we did observe reduced levels of viral spike protein upon TNFα stimulation, whereas IFNβ and IFNγ had no influence. It is unclear why TNFα did reduce viral spike protein load in endothelial cells and limited information is available regarding the effects of TNFα on SARS-CoV-2 virus entry and infection rate. One study reports a link between TNFα and ACE2 expression, which was reduced by systemic anti-TNFα treatment in intestinal cells [26]. However, we did not find a transcriptional regulation of ACE2 in endothelial cells (Suppl. Fig. 6). Thus, one may speculate that TNFα might interfere with other mechanisms resulting in a reduction of viral uptake or destabilization of spike protein.

In conclusion, the lack of a direct cytotoxic or pro-inflammatory effect by SARS-CoV-2 infection of endothelial cells may suggest that the massive endothelial dysfunction and microvascular thrombotic complications observed in patients suffering from COVID-19 is mainly secondarily caused by the inflammatory cascades mediated by the cytokine release syndrome. However, due to reduction of cell number observed after infection of HCAECs with the variant B.1.1.7, patients infected with these SARS-Co-V-2 variants should be closely monitored. Overall, therapeutic interventions aiming at endothelial protection may be warranted to protect against primary or secondary effects to maintain organ integrity during later development of the disease.

## Supplementary Information

Below is the link to the electronic supplementary material.

Supplementary file1 (PDF 1864 KB)

## Abbreviations

ACE2: Angiotensin-converting enzyme 2
D-HPAEC: Human lung pulmonary arterial cells isolated from diabetics
ER: Endoplasmic reticulum
IFN: Interferon
HCAEC: Human coronary artery endothelial cells
HCMVEC: Human cardiac microvascular endothelial cells
HLMVEC: Human lung microvascular endothelial cells
HUVEC: Human umbilical venous endothelial cells
NRP1: Neuropilin-1
SARS-CoV-2: Severe acute respiratory syndrome coronavirus 2
