# Supplementary material for: Increased susceptibility of human endothelial cells to infections by SARS-CoV-2 variants
Source: Basic Res Cardiol. 2021 Jul 5;116(1):42. doi: 10.1007/s00395-021-00882-8 (PMC8256413; doi:10.1007/s00395-021-00882-8)
Supplement: Supplementary file 1 — Supplementary file1 (PDF 1864 KB) [file 395_2021_882_MOESM1_ESM.pdf]

Supplemental Fig. 1

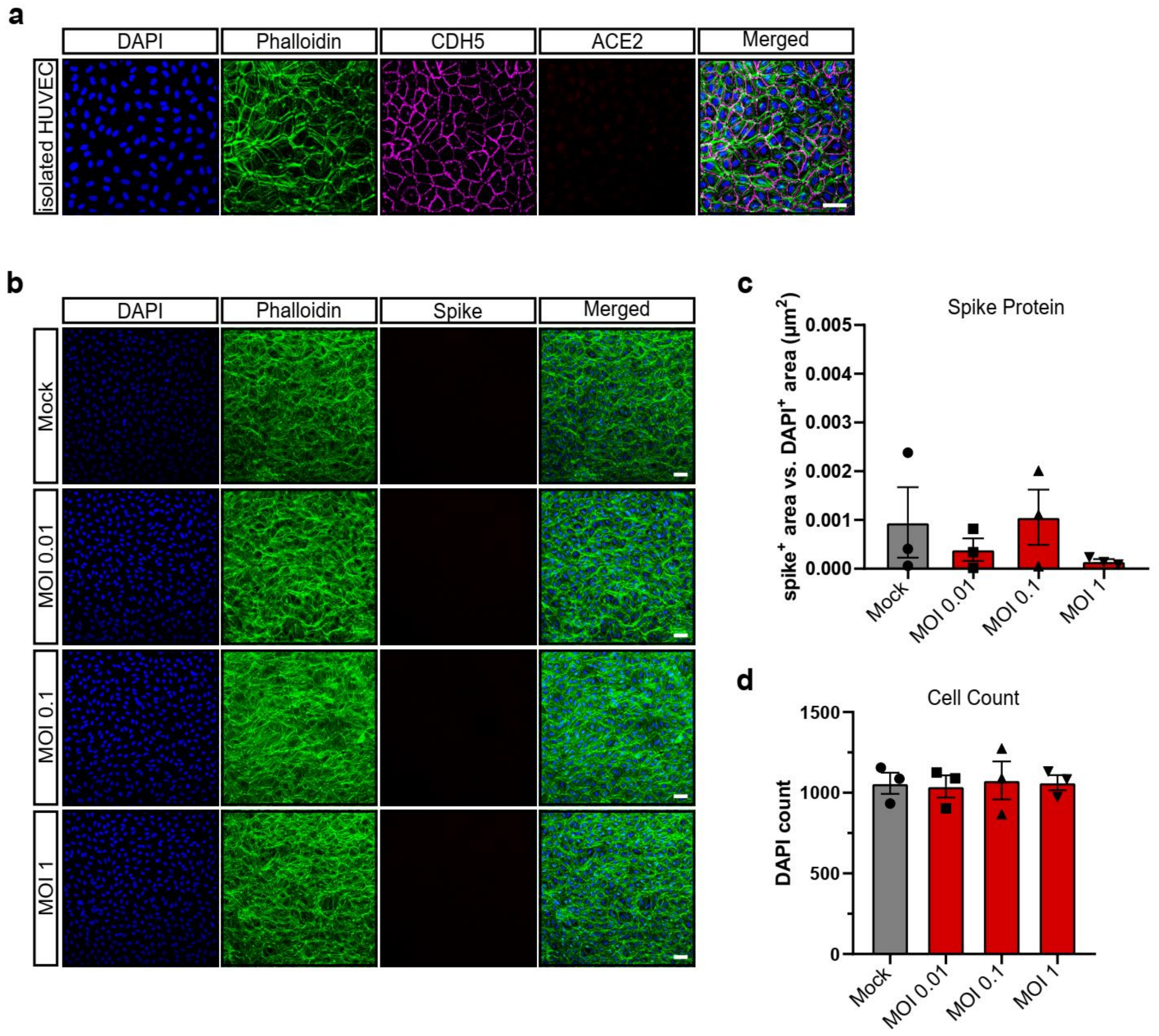

Supplemental Fig. 1: ACE2 and spike expression in isolated HUVEC.
(**a**) Freshly isolated HUVEC were seeded at 80% confluence and stained for ACE2 (red). DAPI (blue), phalloidin (green) and CDH5 (magenta) were used as counter-staining. A representative image is shown. (**b**-**d**) Isolated HUVEC were inoculated dose dependently for 2h with SARS-CoV-2 (MOI 0.01, MOI 0.1, MOI 1) and fixed 5 days post infection. Cells were stained for DAPI (blue), phalloidin (green) and viral spike protein (red, rabbit-SARS-CoV2 Spike primary antibody provided by Hölzel). Spike protein expression was quantified by calculating the ratio of spike-positive vs. DAPI-positive area (panel c) and cell number was determined by counting DAPI-positive nuclei (panel D). Experiments were executed with freshly isolated HUVEC from three different donors. Data are expressed as mean and error bars indicate SEM. After passing normality distribution, data were assessed statistically using ONE-way ANOVA with an post hoc Turkey's multiple comparison test (c, d). n=3. Scale = 50 µm.

Supplemental Fig. 2

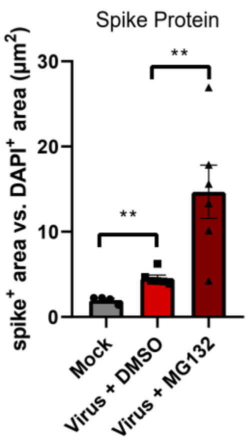

Supplemental Fig. 2: SARS-CoV-2 infection upon proteasomal inhibition. HCAEC were infected in the presence and absence of the proteasomal inhibitor MG132, whereas DMSO served as solvent. Spike protein was detected using immunofluorescence imaging (n=4). Statistical power was determined using an unpaired ttest to compare infected cells to the mock control.

Supplemental Fig. 3

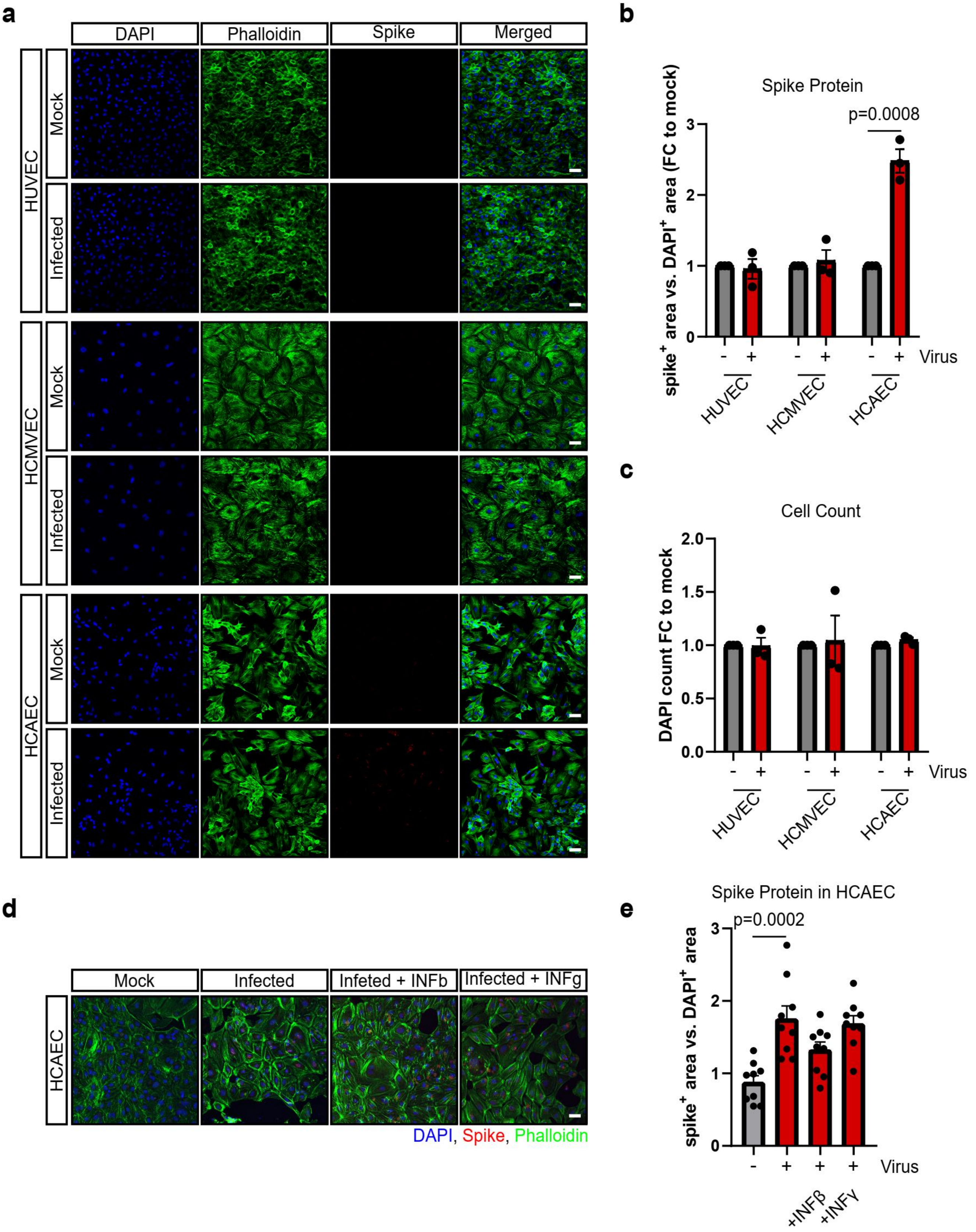

Supplemental Fig. 3: TNFα attenuates viral infection in HCAEC. (**a**) Human umbilical venous endothelial cells (HUVEC), human coronary artery endothelial cells (HCAEC), human cardiac microvascular endothelial cells (HCMVEC), and human lung microvascular endothelial cells (HLMVEC), and human lung pulmonary arterial cells isolated from diabetics (D-HPAEC) were purchased from Lonza and were treated with 30 ng/mL TNFα prior to SARS-CoV-2 inoculation (as described in Fig. 2). Cells were cultured for 5 days and fixed with 4% PFA. Spike protein (red) was detected by using the rabbit-SARS-CoV2 Spike primary antibody (provided by Hölzel) and cells were counterstained for DAPI (blue) and phalloidin (green). (**b**) Quantification of data shown in a. (**c**) DAPI positive cells were counted in experiments shown in a. Data are shown as mean and error bars indicate the standard error of the mean (SEM). (**d-e**) HCAEC were infected with SARS-CoV-2 isolates in the presence and absence of 10 ng/mL human recombinant interferon beta (INFb) or interferon gamma (INFg). Cells were cultured for 5 days and fixed with 4% PFA. Spike protein (red) was detected by using the rabbit-SARS-CoV2 Spike primary antibody (provided by Hölzel) and cells were counterstained for DAPI (blue) and phalloidin (green). After passing normality tests, data were statistically accessed by using an unpaired, two-sided T-test to compare mock treated cells to their respective infected counterpart (a-c). Multi-group comparison was performed by One-way ANOVA with an post-hoc Turkey's test. (d-e) All experiments were conducted with at least n = 3 (a-c) or n=9 (d-e). Scale bars = 50 µm.

Supplemental Fig. 4

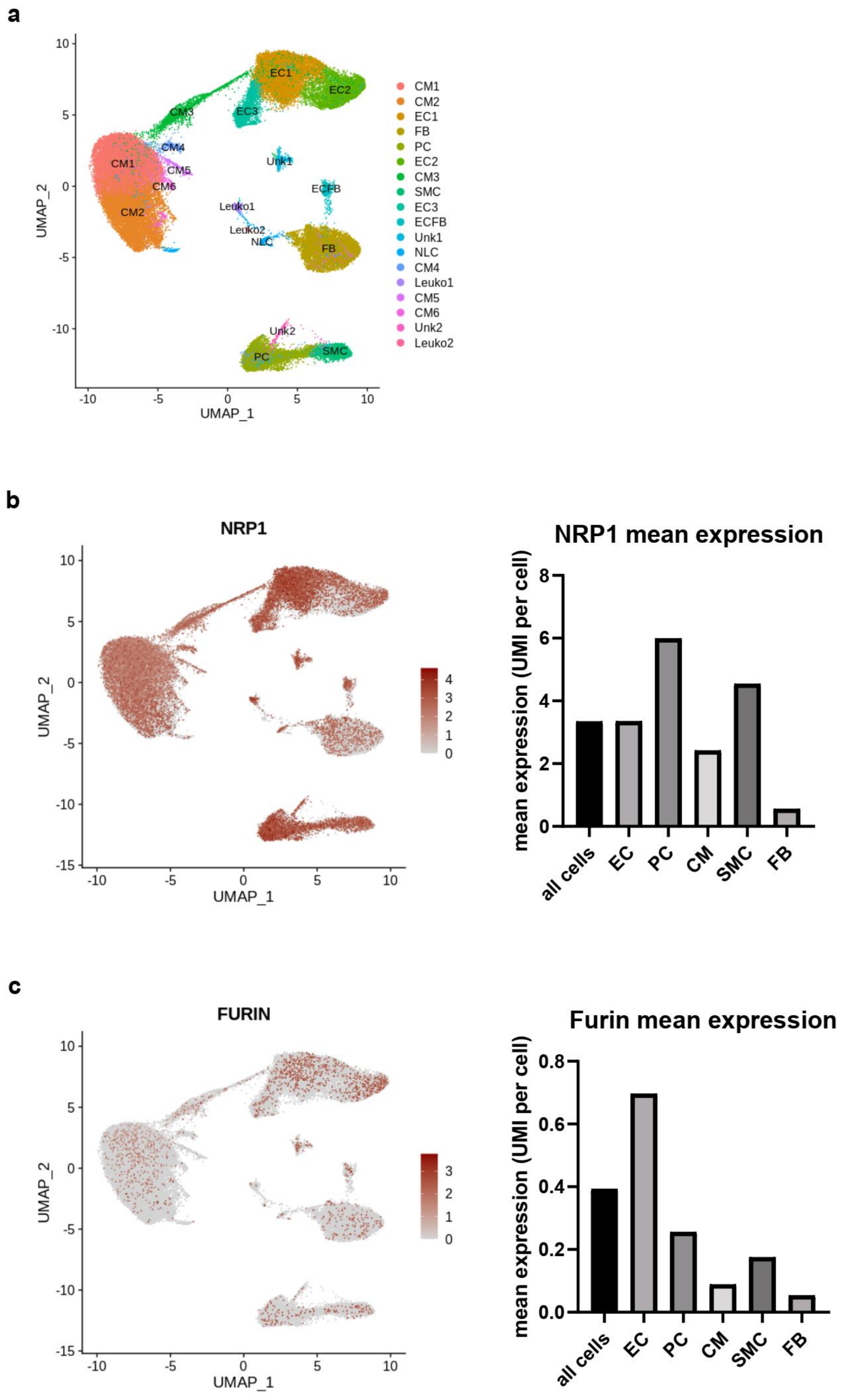

Supplemental Fig. 4: Analysis of single nuclei RNA sequencing of heart tissue using a published data set. (**a**) Cell annotation and NRP1 (**b**) and FURIN (**c**) expression in different cell types. EC=endothelial cells.

Supplemental Fig. 5

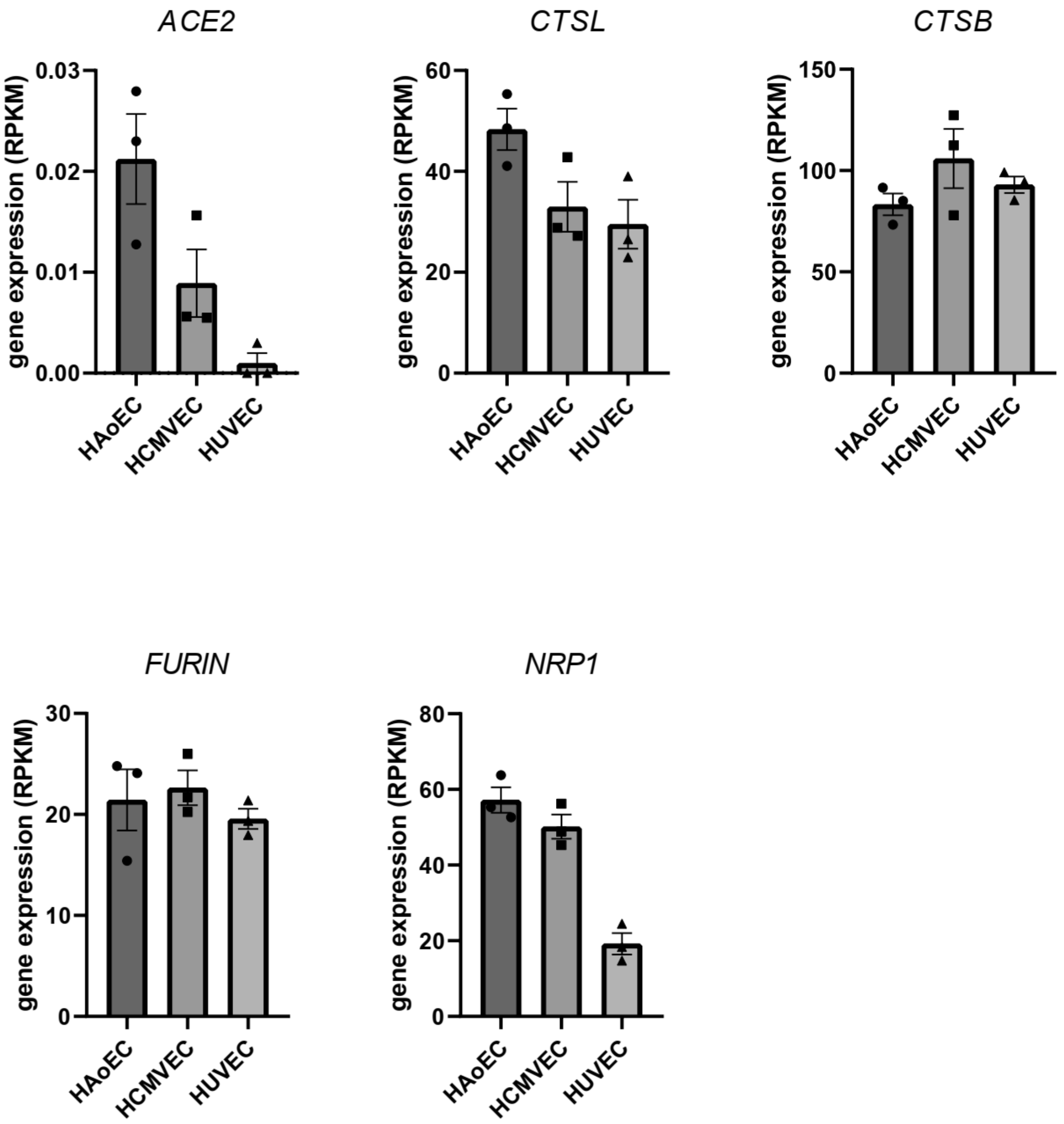

Supplemental Fig. 5: Analysis of bulk RNA sequencing human aortic EC, human cardiac microvascular EC and HUVEC. Expression of factors involved in SARS-CoV-2 entry. Data were received from bulk RNA sequencing data of human aortic EC (HAoEC), human cardiac microvascular EC (HCMVEC) and HUVEC. n=3.

Supplemental Fig. 6

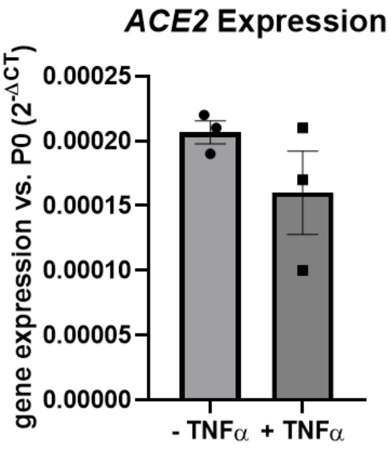

Supplemental Fig. 6: ACE2 expression in TNFα-treated HCAEC. HCAEC were stimulated for 24h with 30 ng/mL TNFα and ACE2 mRNA expression was detected by RT-qPCR. Data are shown as mean and error bars indicate SEM (n=3).

Supplemental Tab. 1

Supplemental Tab. 1: Antibodies and reagent used for immunostaining.

Antibody / Reagent	Product-Code
Monoclonal Anti-α-Actinin (Sarcomeric actin) antibody produced in mouse (1:300)	A7811, Sigma-Aldrich
Polyclonal Human ACE-2 Antibody produced in goat (1:100)	AF933, R&D Systems.
Monoclonal SARS-CoV-2 (2019-nCoV) Spike S1 Antibody, produced in rabbit (1:1500 / 1:100)	40150-R007, Sino Biological
Monoclonal antibody directed against dsRNA, produced in mouse (1:150)	10010500, SCICONS J2, English & Scientific Consulting Kft., Szirák, Hungary
Polyclonal anti-CDH5 antibody, produced in rabbit (1:100)	2500S, Cell Signaling Technologies
Monoclonal anti-Calnexin Antibody (AF18), produced in mouse	MA3-027, ThermoFisher Scientific
Donkey anti-Rabbit IgG (H+L) Highly Cross-Adsorbed Secondary Antibody, Alexa Fluor 555 (1:200)	A-31572, Invitrogen
Donkey anti-Mouse IgG (H+L) Highly Cross-Adsorbed Secondary Antibody, Alexa Fluor Plus 647 (1:200)	A32787, Invitrogen
Donkey anti-Goat IgG (H+L) Cross-Adsorbed Secondary Antibody, Alexa Fluor 555 (1:200)	A-21432, Invitrogen
Alexa Fluor™ 488 Phalloidin (1:100)	A12379, Invitrogen
Ulex Europaeus Agglutinin I (UEA I), Biotinylated (1:50)	B-1065-2, Vector Laboratories
DAPI (1:1000)	62248, Thermo Scientific™
Streptavidin, Alexa Fluor™ 405 (1:100)	S32351, Invitrogen

Supplemental Tab. 2

Supplemental Tab. 2: List of primers used for PCR.

hRPLP0 -F: 5‘-TCGACAATGGCAGCATCTAC-3‘
hRPLP0-R: 5‘-ATCCGTCTCCACAGACAAGG-3‘
TMPRSS2-F: 5‘-CTGCCAAGGTGCTTCTCATT-3‘
TMPRSS2-R: 5‘-CTGTCACCCTGGCAAGAATC-3‘
TMPRSS4-F: 5‘-CCAAGGACCGATCCACACT-3‘
TMPRSS4-R: 5‘-GTGAAGTTGTCGAAACAGGCA-3‘
CTSL-F: 5’-AGGAGAGCAGTGTGGGAGAA-3‘
CTSL-R: 5’-ATCTGGGGGCCTCATAAAAC-3‘
CTSB-F: 5’-CCAGGGAGCAAGACAGAGAC-3‘
CTSB-R: 5’-GAGACTGGCGTTCTCCAAAG-3‘
Furin-F: 5’-GCCACATGACTACTCCGCAGAT-3‘
Furin-R: 5’-TACGAGGGTGAACTTGGTCAGC-3‘
CD209L-F: 5’-CAGCGGGAAAACATGAGTGAC-3‘
CD209L-R: 5’-GGGACCTTGGACACTTGGAC-3‘
IL-6-F: 5’- GCAGAAAAAGGCAAAGAATC-3’
IL-6-R: 5’- CTACATTTGCCGAAGAGC-3’
VCAM1-F: 5’- GGGAAGCCGATCACAGTCAA-3’
VCAM1-R: 5’- CTCCAGCCTGTCAAATGGGT-3’
ICAM1-F: 5’- GAGCTTCGTGTCCTGTATGG-3’
ICAM1-R: 5’- TTTCTGGCCACGTCCAGTTT-3’
VEGF-F: 5’- CCCTGATGAGATCGAGTACA-3’
VEGF-R: 5’- AGCAAGGCCCACAGGGATTT-3’
NRP1-F: 5’- GGATCACACAGGAGATGGCA-3’
NRP1-R: 5’- GCTGATCGTACTCCTCTGGC-3’
RdRP_SARSr-F: 5’-GTGARATGGTCATGTGTGGCGG-3’
RdRP_SARSr-R: 5’-CARATGTTAAASACACTATTAGCATA-3’
EDEM1-F: 5’-CGAGTTCCAGAAAGCCGTCA-3’
EDEM1-R: 5’-GGGCTGCTTGGAGTCAGTTA-3’
BiP-F: 5’-TGGAGGTGGGCAAACAAAGA-3’
BiP1-R: 5’-ACAACTGCATGGGTAACCTTCT-3’
ATF4-F: 5’-TCCAACAACAGCAAGGAGGA-3’
ATF4-R: 5’-ACGTGGTCAGAAGGTCATCT-3’
DDIT3-F: 5’-GCTGGAACCTGAGGAGAGAG-3’
DDIT3-R: 5’-TGCTTTCAGGTGTGGTGATG-3’

Supplemental Tab. 3

Supplemental Tab. 3: Determination of viral titer from infected endothelial cell supernatant. Infectious virus in supernatants from infected endothelial cells was determined by titration in CaCo2 cells 48 h post infection.

	HUVEC	HCMVEC	HCAEC	HLMVEC	D-HPAEC	CaCo2
TCID50/mL	0	0	0	0	0	$3*10^7$
